# New infrastructure to support clinical translational research at the US National Institutes of Health: role of the NIH Clinical Center

**DOI:** 10.1186/1479-5876-10-S2-A2

**Published:** 2012-10-17

**Authors:** John I Gallin

**Affiliations:** 1 Clinical Center, National Institutes of Health, Bethesda, Maryland 20892, USA

## 

The development of new drugs and devices is expensive with long timelines and high rate of failure. The director of NIH is concerned with these issues and has made reengineering the translational sciences a top priority [1]. One outcome of the planning has been the creation of a new NIH National Center for Advancing Translational Sciences (NCATS) [2]. NCATS has three major goals: reengineering translational sciences, creating a network of clinical and translational science awardees (CTSAs) at 60 academic institutions in the US and supporting research on rare diseases and therapeutics. Recent novel approaches to technology development under NCATS leadership include designing new “organ chips” and use of induced pluripotent stem cells (iPS cells) to screen for drug toxicity. Another project is the 1000 genomes project which estimated that human genomes typically contain about 100 genuine loss of function variants with about 20 genes completely inactivated but without obvious disease consequence. In some cases the loss of function variants are associated but with protection from disease thereby providing new targets for drug discovery [3]. Another effort to reengineer drug discovery is a drug repurposing project that has resulted in partnerships with 8 pharmaceutical companies who recently announced 58 compounds available for repurposing studies [4]. The Office of Rare Diseases Research has moved into NCATS and is working to establish partnerships throughout the world for the study of rare diseases. The Office of Rare Diseases Research continues to work on the development of novel treatment strategies. The NIH Clinical Center, the largest hospital in the world totally dedicated to clinical research, will work closely with NCATS. A new vision for the NIH Clinical Center will be “to open its doors” to outside investigators through new partnerships between extramural scientists and investigators in the NIH intramural program. These partnerships will include access to the special technical resources at the Clinical Center, access to the clinical research training programs including the NIH curriculum for clinical research training, courses designed to demystify medicine for PhDs, as well as a sabbatical in clinical research management. In addition, the Clinical Center will make available new tools it has developed for protocol management and information technology (see http://www.cc.nih.gov/ for review of the Clinical Center programs). The many approaches NIH is implementing to improve translational research will be monitored closely for success.
